# Supplementary material for: New land-use change scenarios for Brazil: Refining global SSPs with a regional spatially-explicit allocation model
Source: PLoS One. 2022 Apr 20;17(4):e0256052. doi: 10.1371/journal.pone.0256052 (PMC9020719; doi:10.1371/journal.pone.0256052)
Supplement: S1 Appendix — (PDF) [file pone.0256052.s001.pdf]

SUPPORTING INFORMATION

Bezerra *et al*. (2022) New land change scenarios for Brazil: refining the global SSPs with regional spatially-explicit allocation model.

Appendix S1 - LuccMEBR: scenario-dependent spatiotemporal drivers

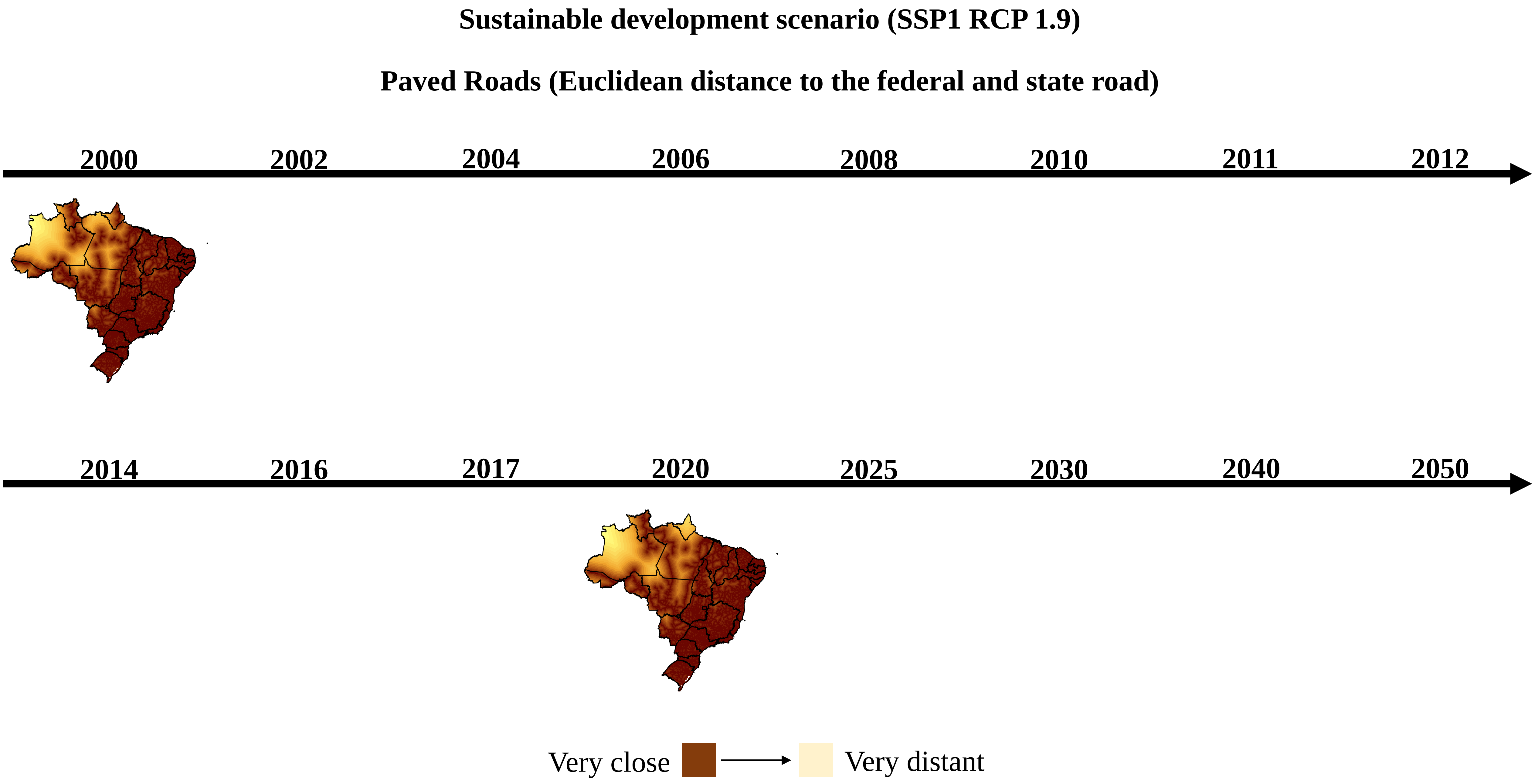

The source of States boundaries is according to IBGE [31]

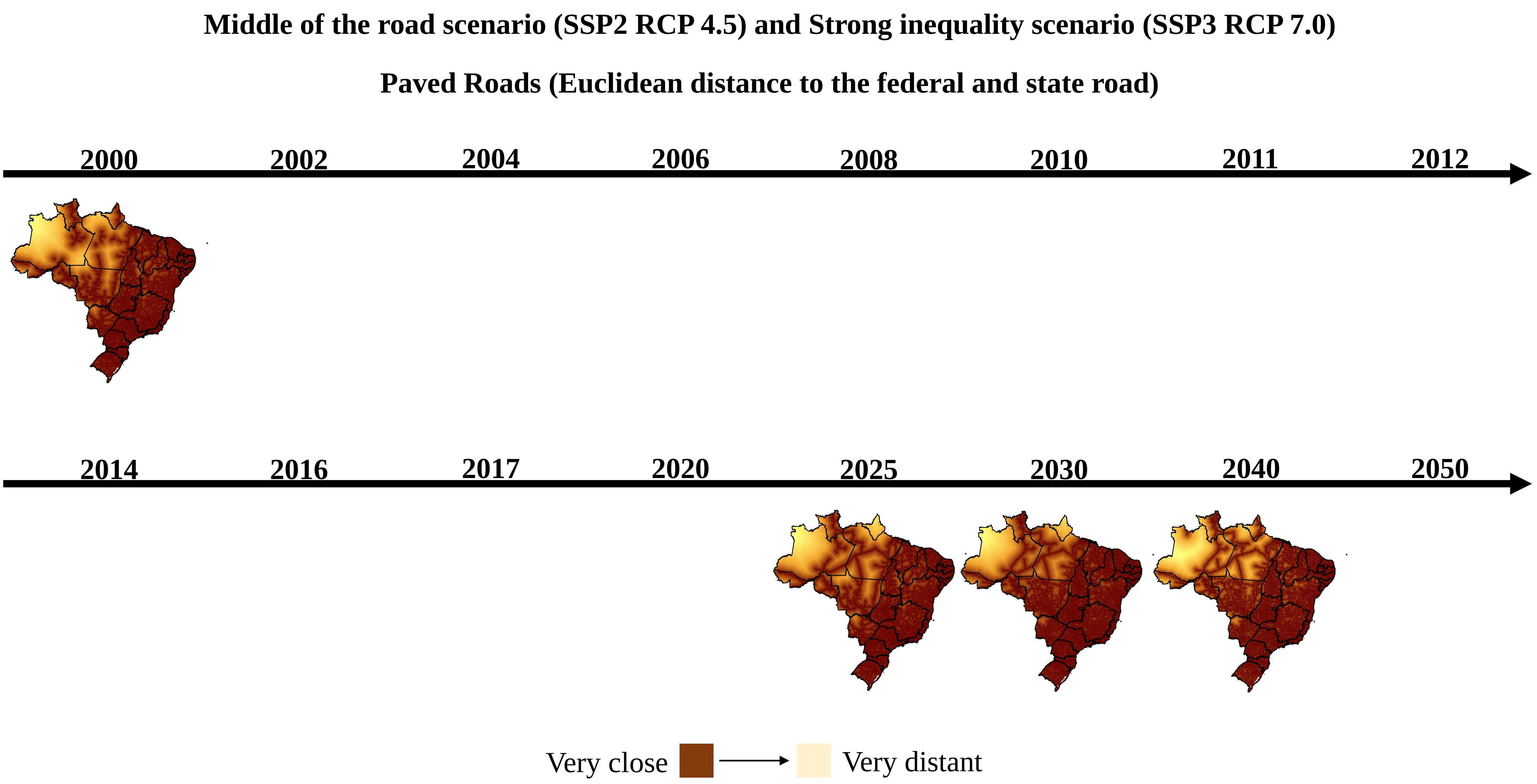

The source of States boundaries is according to IBGE [31]

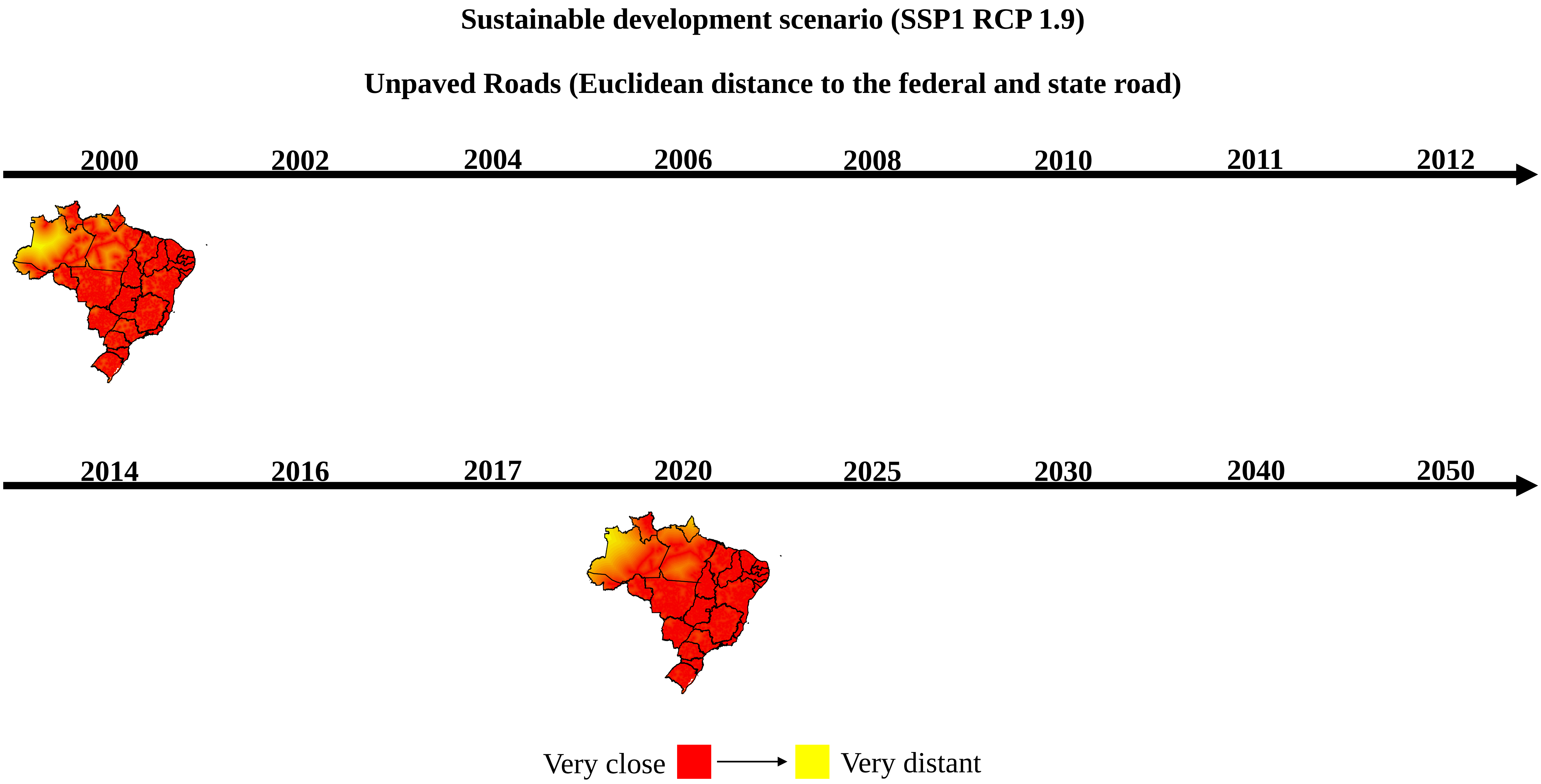

The source of States boundaries is according to IBGE [31]

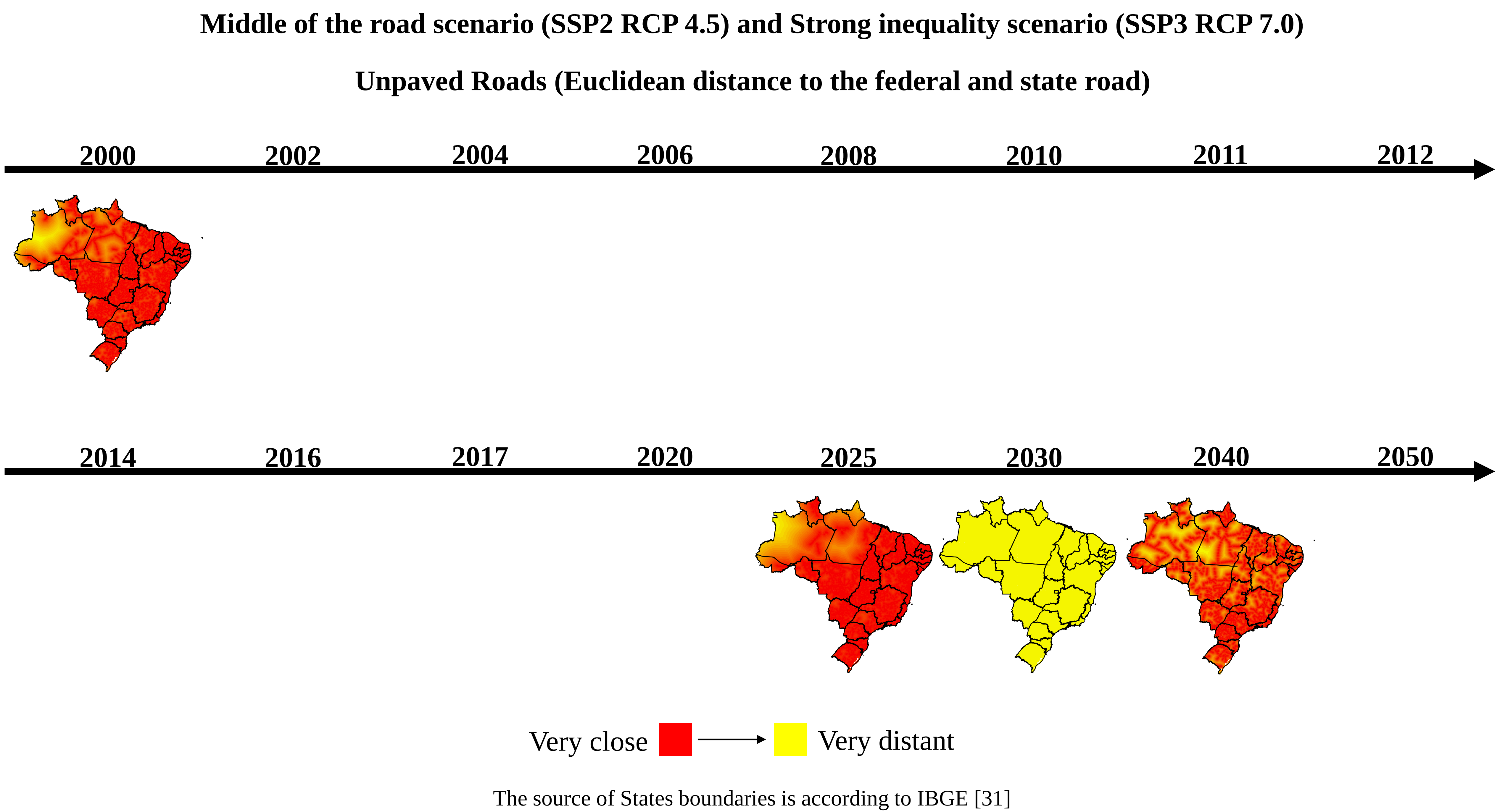
Middle of the road scenario (SSP2 RCP 4.5) and Strong inequality scenario (SSP3 RCP 7.0)
Unpaved Roads (Euclidean distance to the federal and state road)
2000
2002
2004
2006
2008
2010
2011
2012
2014
2016
2017
2020
2025
2030
2040
2050
Very close
Very distant
The source of States boundaries is according to IBGE [31]

Sustainable development scenario (SSP1 RCP 1.9)

National Markets (Connectivity index via the road network to São Paulo or Recife)

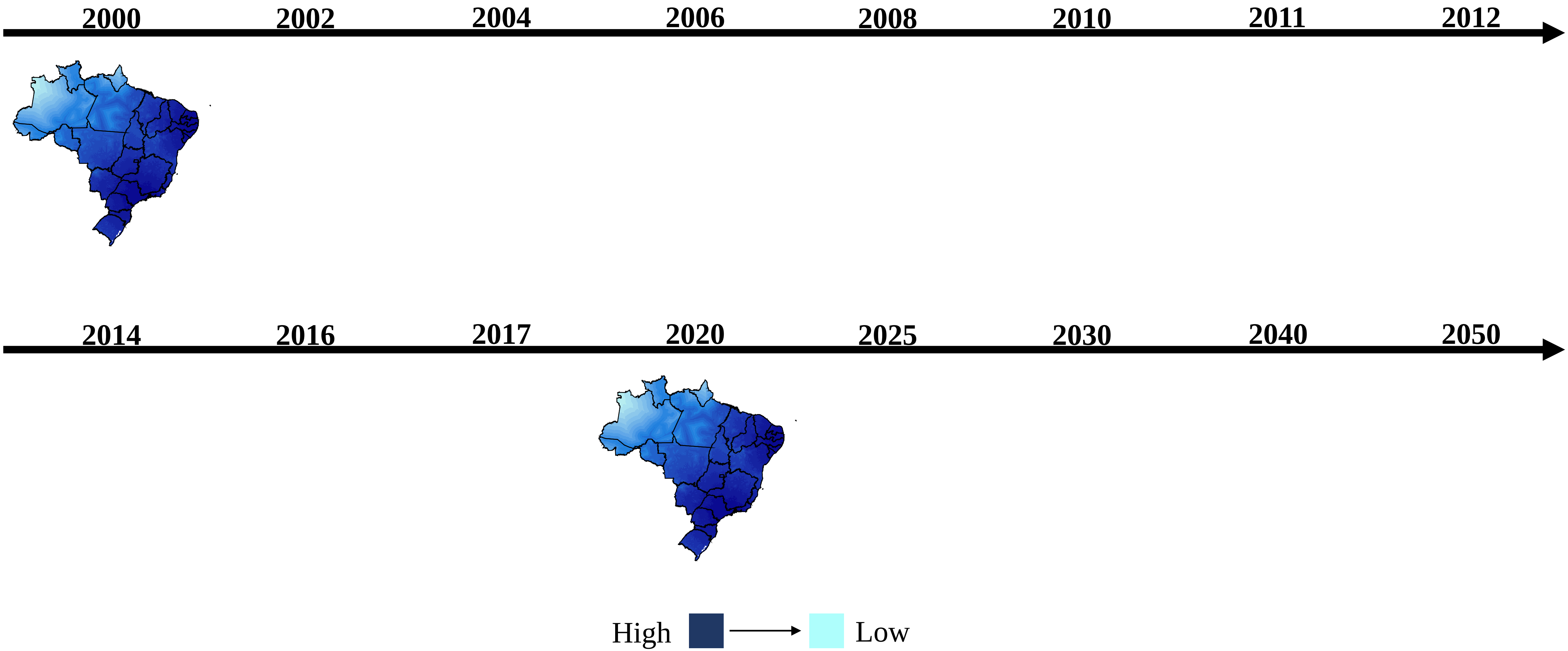

The source of States boundaries is according to IBGE [31]

Middle of the road scenario (SSP2 RCP 4.5) and Strong inequality scenario (SSP3 RCP 7.0)

National Markets (Connectivity index via the road network to São Paulo or Recife)

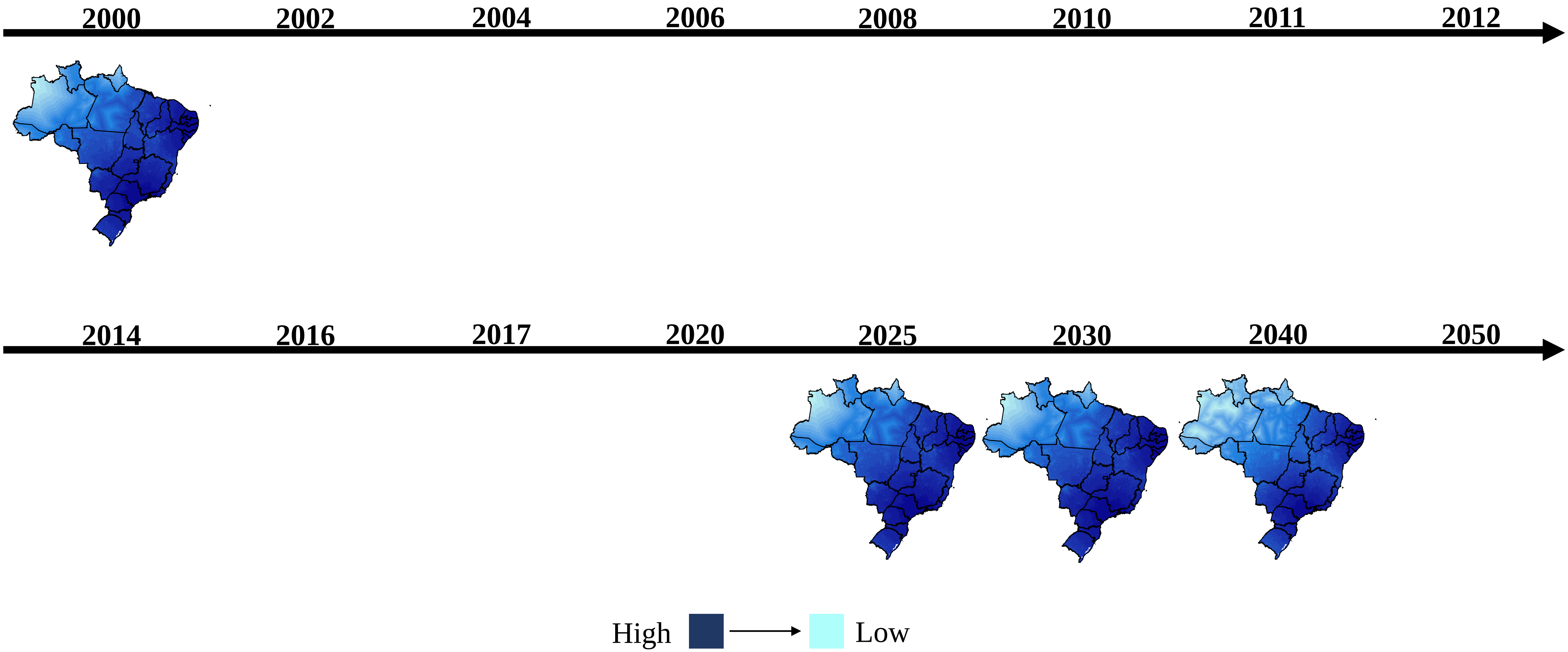

The source of States boundaries is according to IBGE [31]

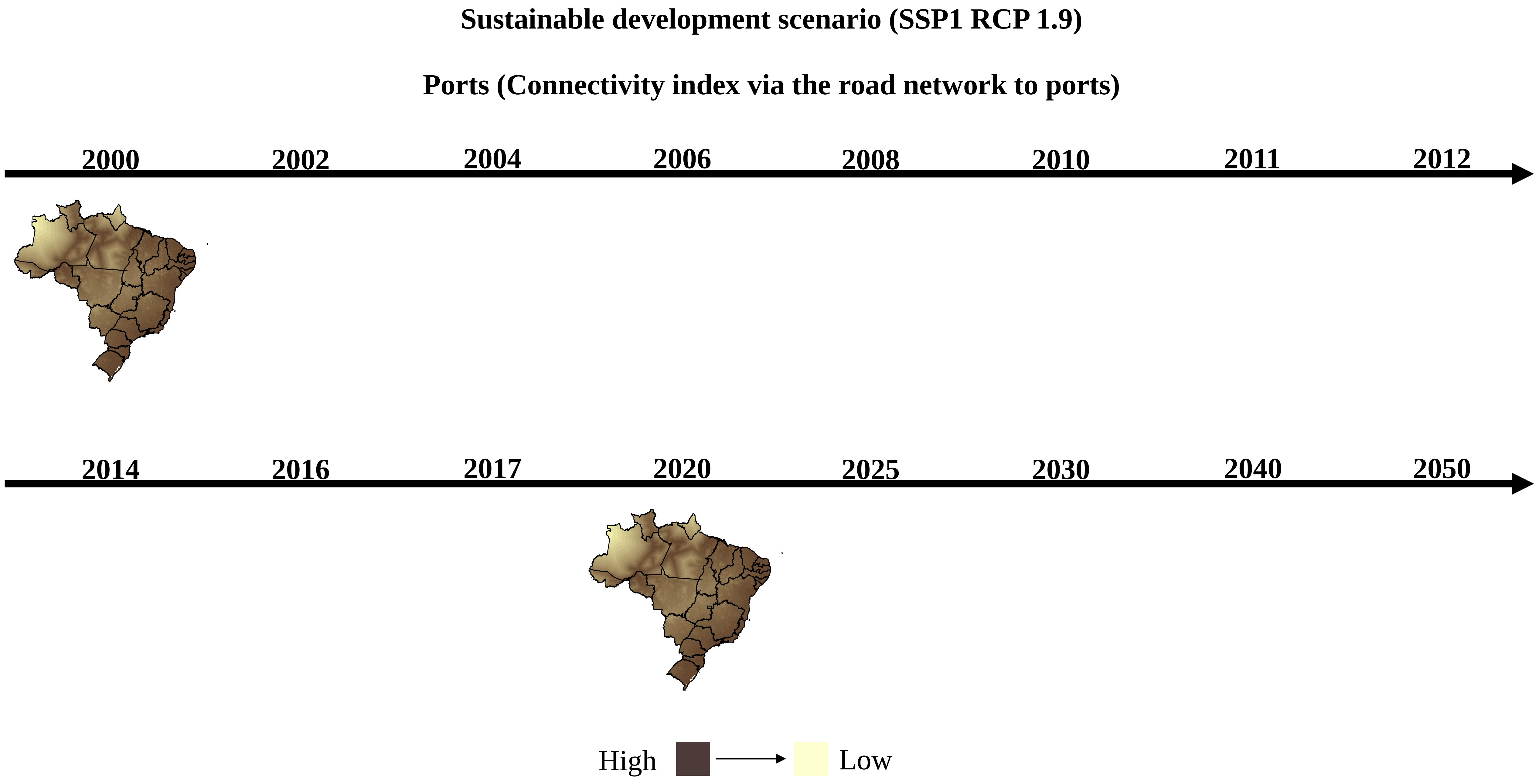

The source of States boundaries is according to IBGE [31]

Middle of the road scenario (SSP2 RCP 4.5) and Strong inequality scenario (SSP3 RCP 7.0)

Ports (Connectivity index via the road network to ports)

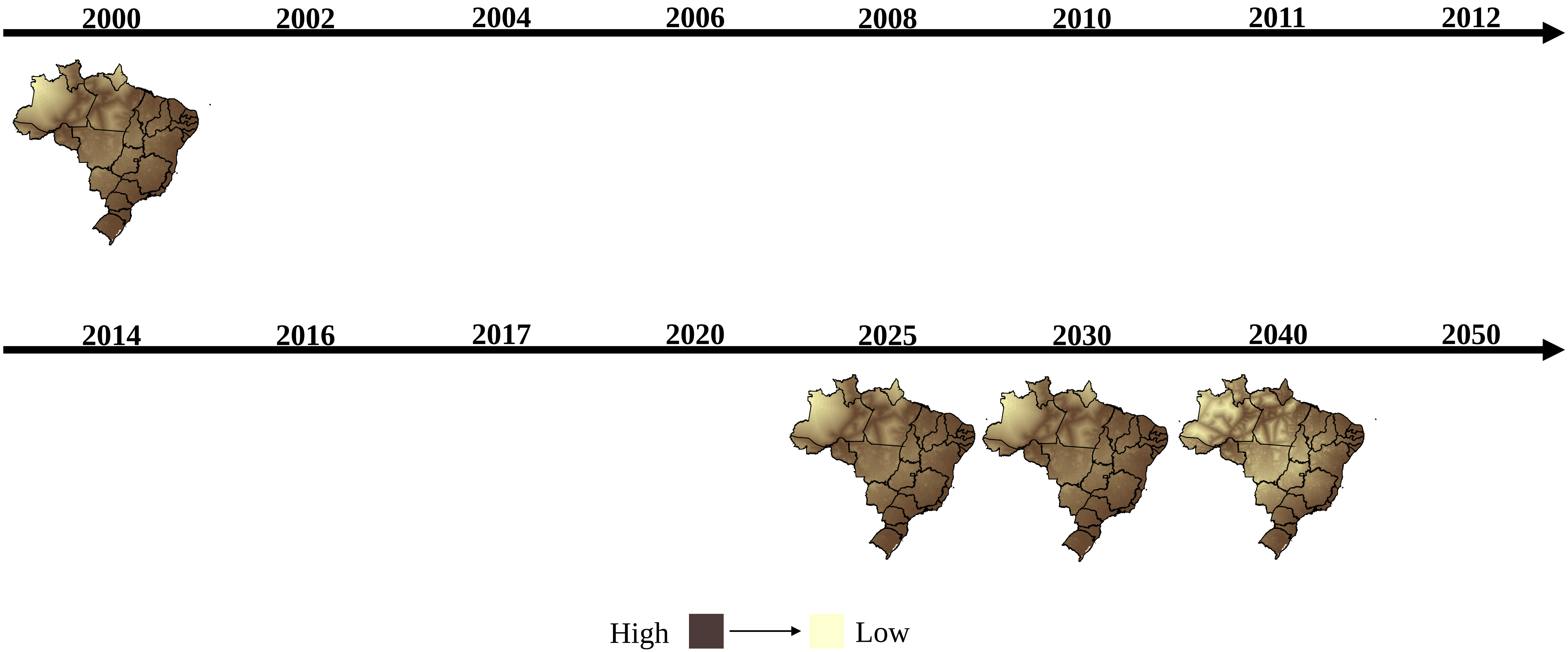

The source of States boundaries is according to IBGE [31]

Sustainable development scenario (SSP1 RCP 1.9) and Middle of the road scenario (SSP2 RCP 4.5)

Agricultural Settlements (Percentage of agricultural settlements without sustainable use settlements)

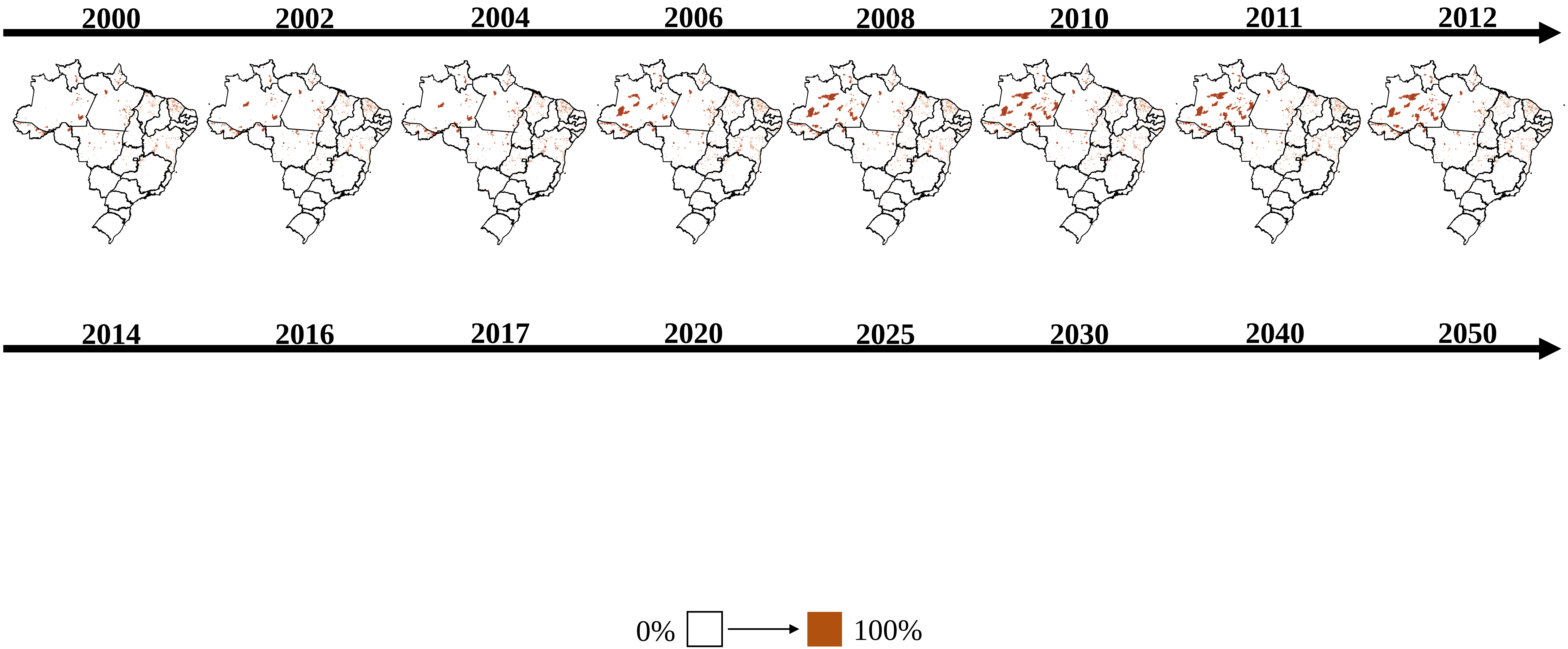

The source of States boundaries is according to IBGE [31]

Strong inequality scenario (SSP3 RCP 7.0)

Agricultural Settlements (Percentage of agricultural settlements without sustainable use settlements)

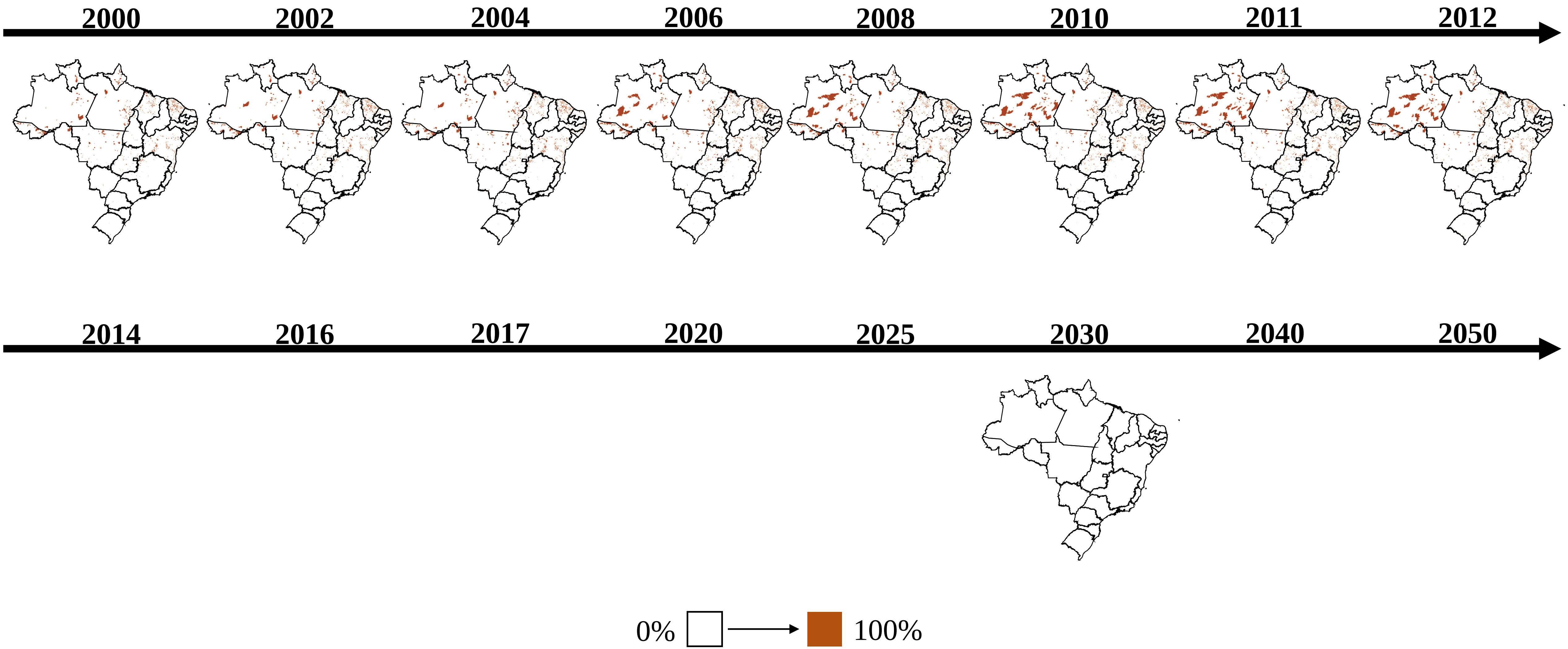

The source of States boundaries is according to IBGE [31]

Sustainable development scenario (SSP1 RCP 1.9) and Middle of the road scenario (SSP2 RCP 4.5)

Protected Areas (Percentage of cell area covered by protected areas: All (Ucs integral protection, TIs, AM))

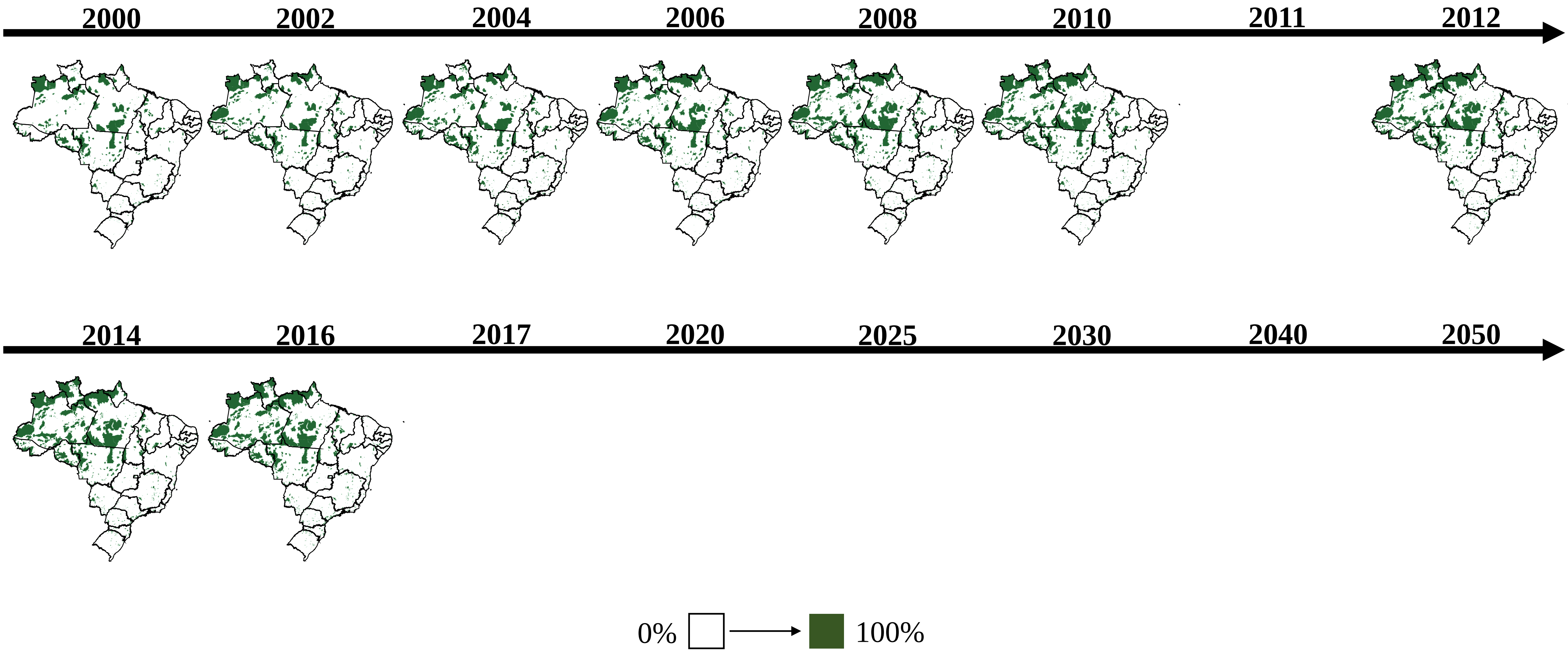

The source of States boundaries is according to IBGE [31]

Strong inequality scenario (SSP3 RCP 7.0)

Protected Areas (Percentage of cell area covered by protected areas: All (Ucs integral protection, TIs, AM))

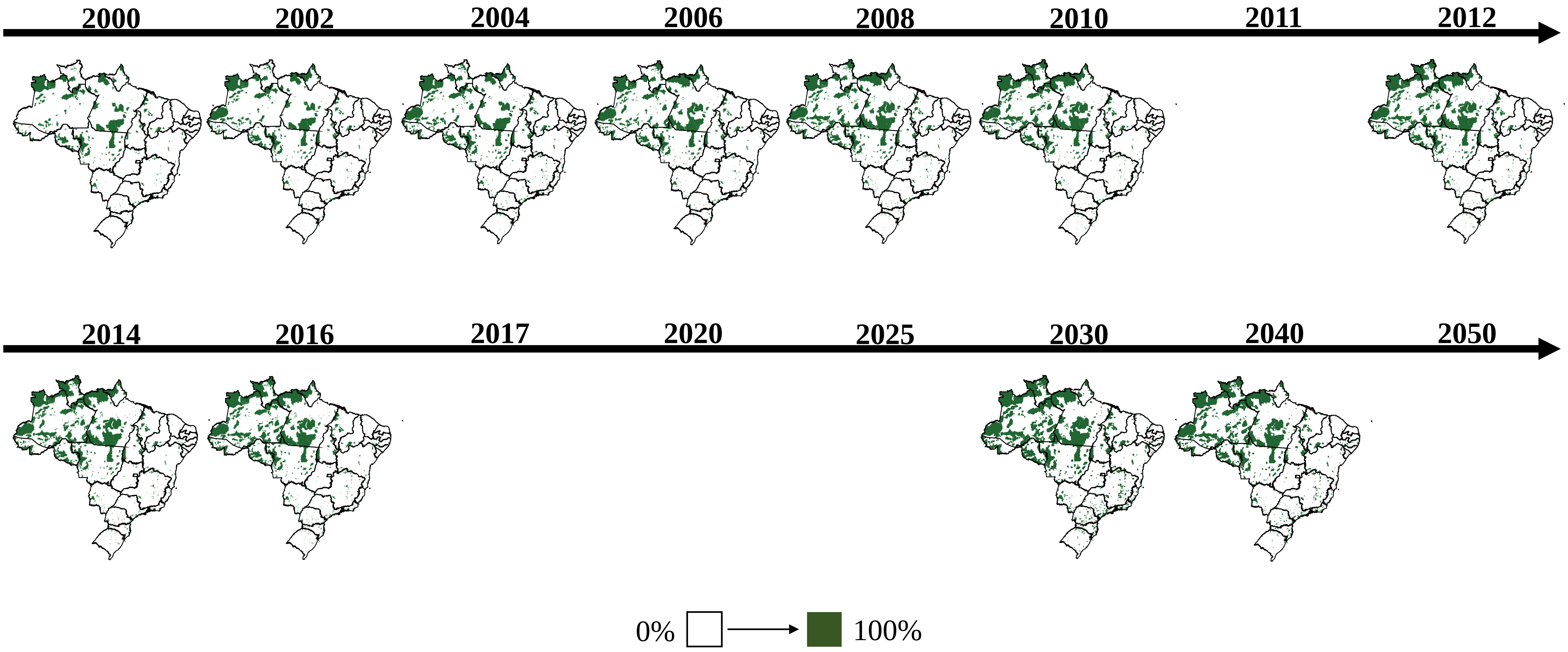

The source of States boundaries is according to IBGE [31]

Scenario independent factors

Agricultural Establishments (Percentage of areas of agricultural establishments with 100 ha)

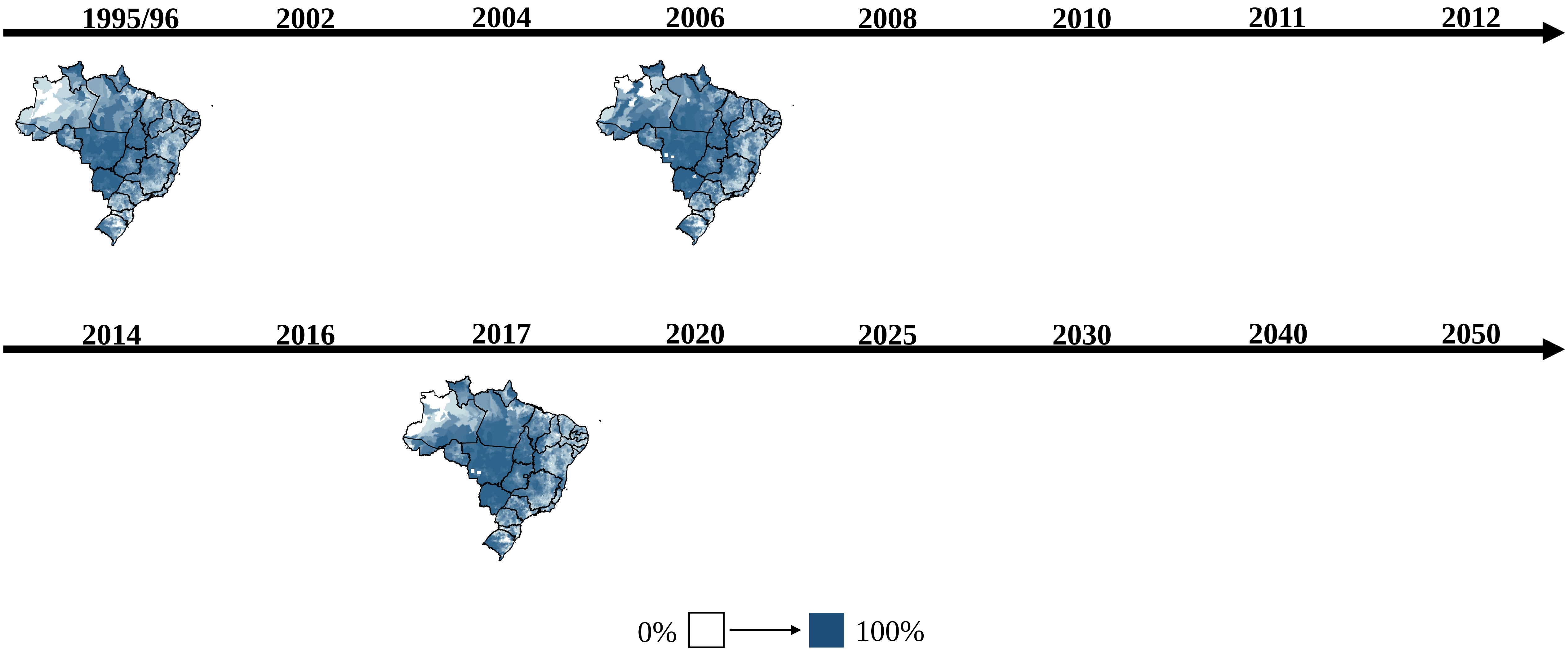

The source of States boundaries is according to IBGE [31]

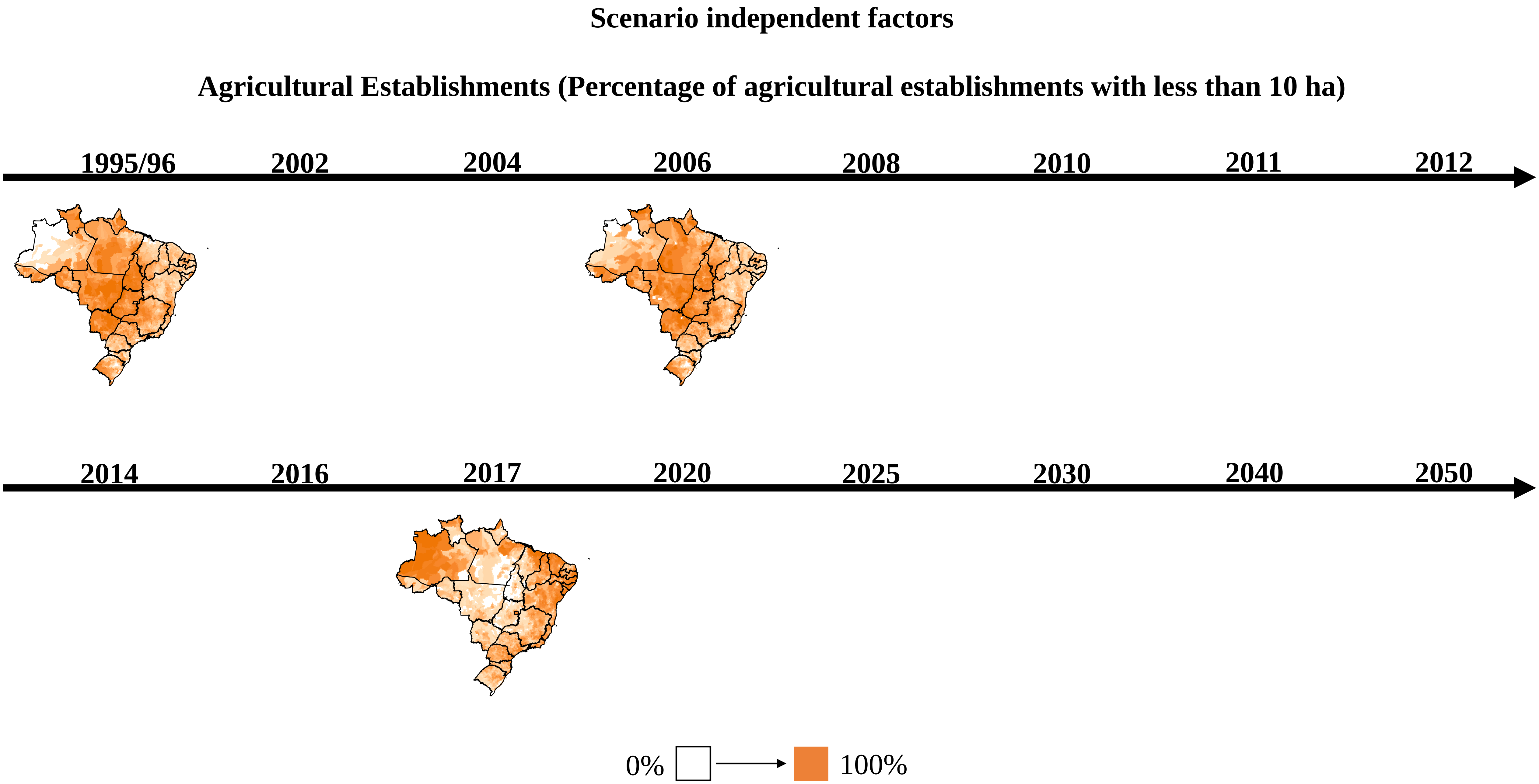

The source of States boundaries is according to IBGE [31]

Scenario independent factors

Agricultural Establishments (Percentage of agricultural establishments with 10 to less than 100 ha)

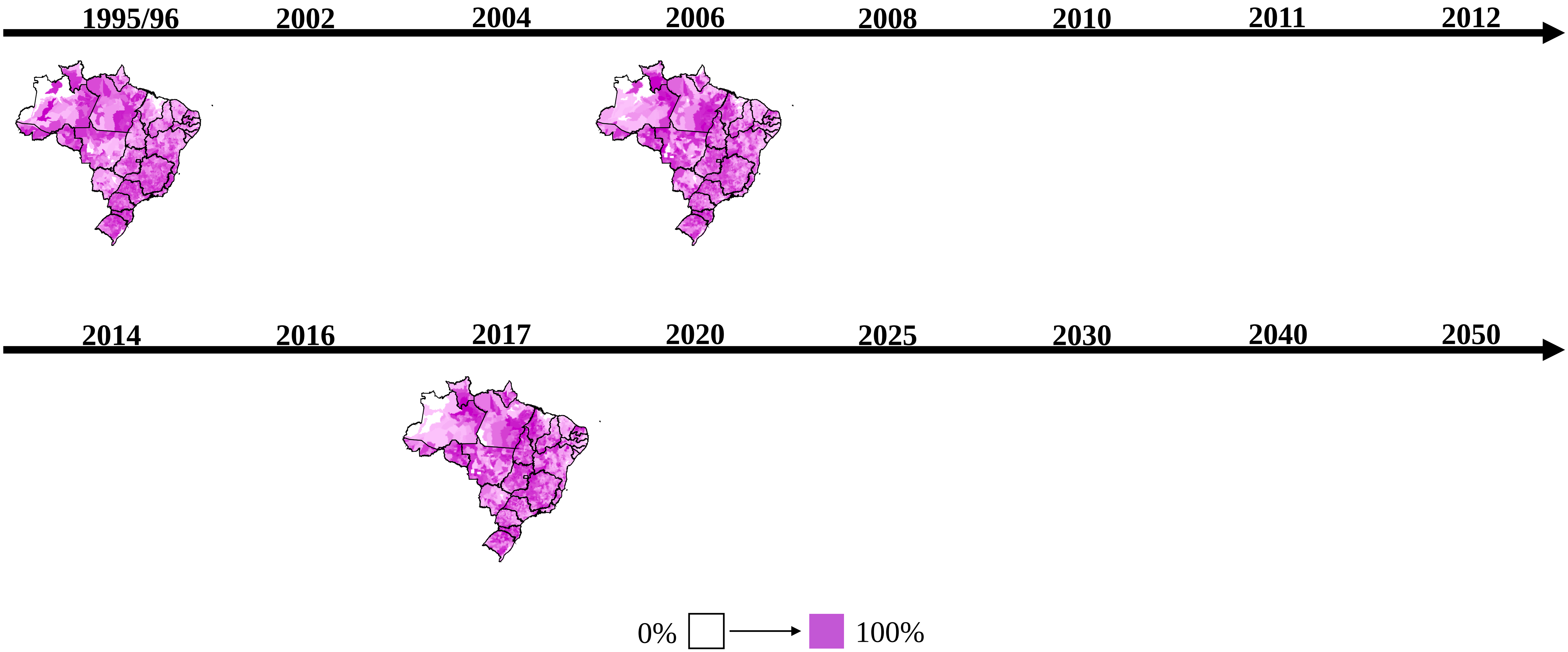

The source of States boundaries is according to IBGE [31]

Scenario independent factors

Agricultural Establishments (Percentage of areas of agricultural establishments with 100 ha more)

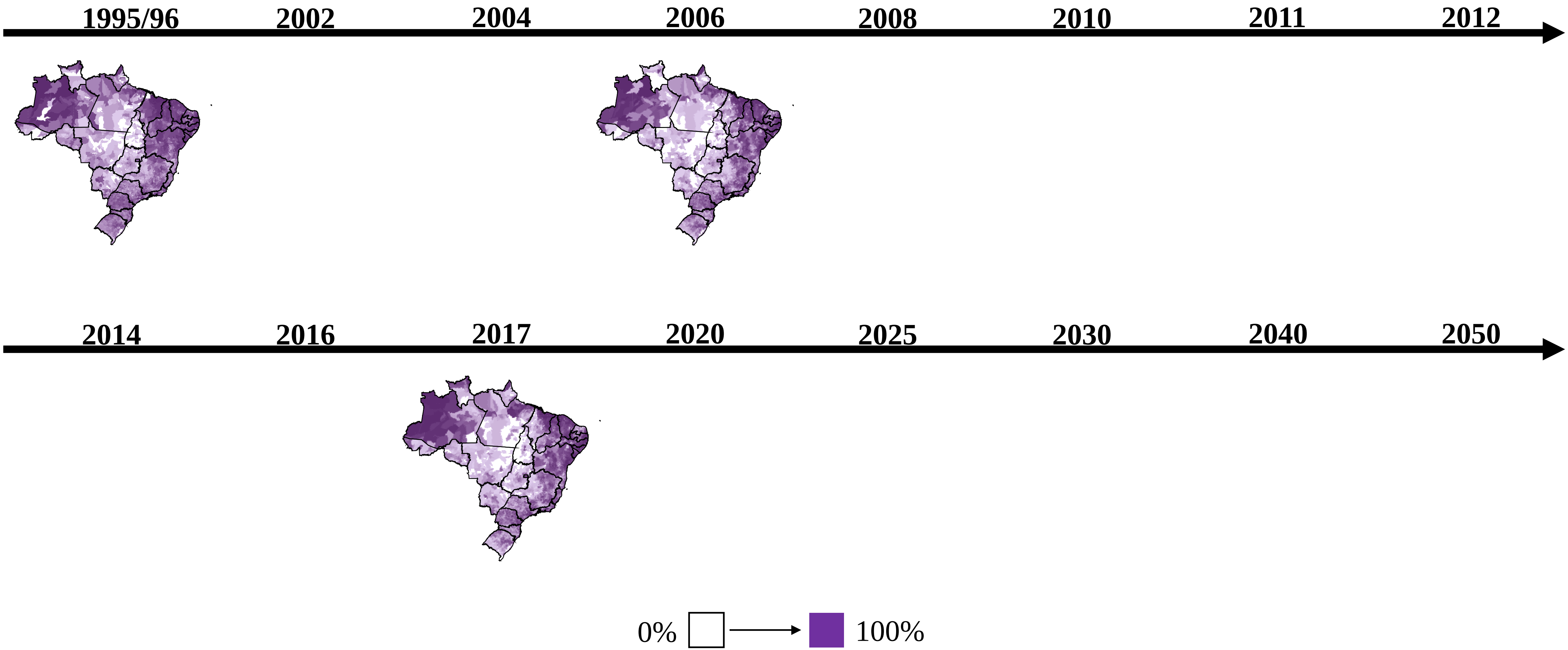

The source of States boundaries is according to IBGE [31]

Scenario independent factors

Slope (Percentage of cell area covered by slope more than 3 to 8%)

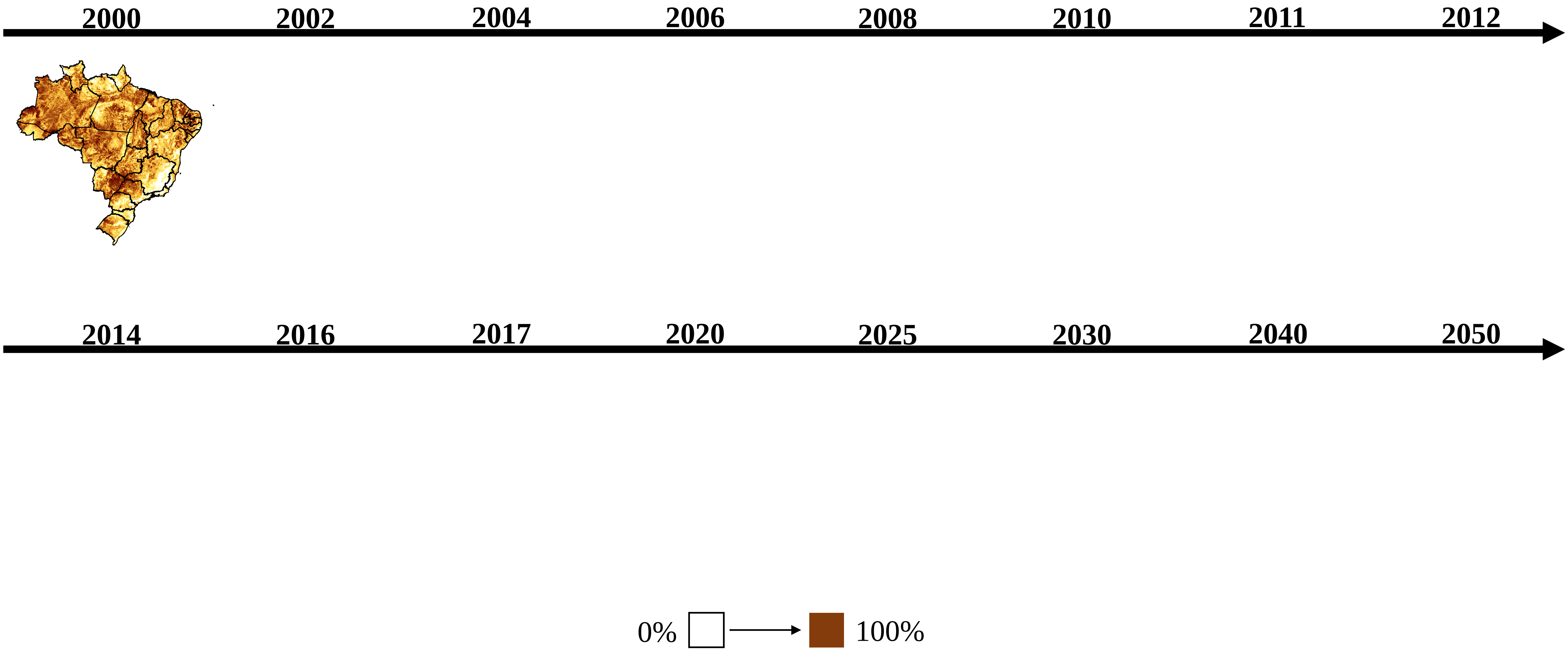

The source of States boundaries is according to IBGE [31]

Scenario independent factors

Slope (Percentage of cell area covered by slope more than 8 to 13%)

2000 2002 2004 2006 2008 2010 2011 2012

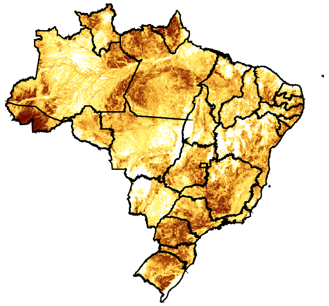

2014 2016 2017 2020 2025 2030 2040 2050

0% → 100%

The source of States boundaries is according to IBGE [31]

Scenario independent factors

Slope (Percentage of cell area covered by slope more than 13 to 20%)

2000 2002 2004 2006 2008 2010 2011 2012

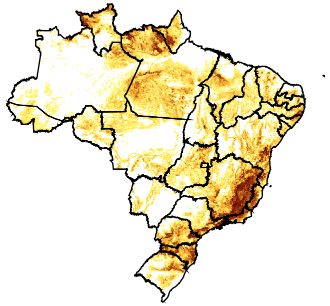

2014 2016 2017 2020 2025 2030 2040 2050

0% → 100%

The source of States boundaries is according to IBGE [31]

Scenario independent factors

Slope (Percentage of cell area covered by slope more than 20 to 45%)

2000 2002 2004 2006 2008 2010 2011 2012

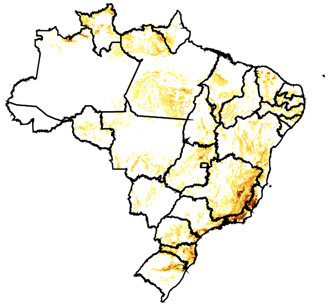

2014 2016 2017 2020 2025 2030 2040 2050

0% → 100%

The source of States boundaries is according to IBGE [31]

Scenario independent factors

Rivers (Euclidean distance to the rivers)

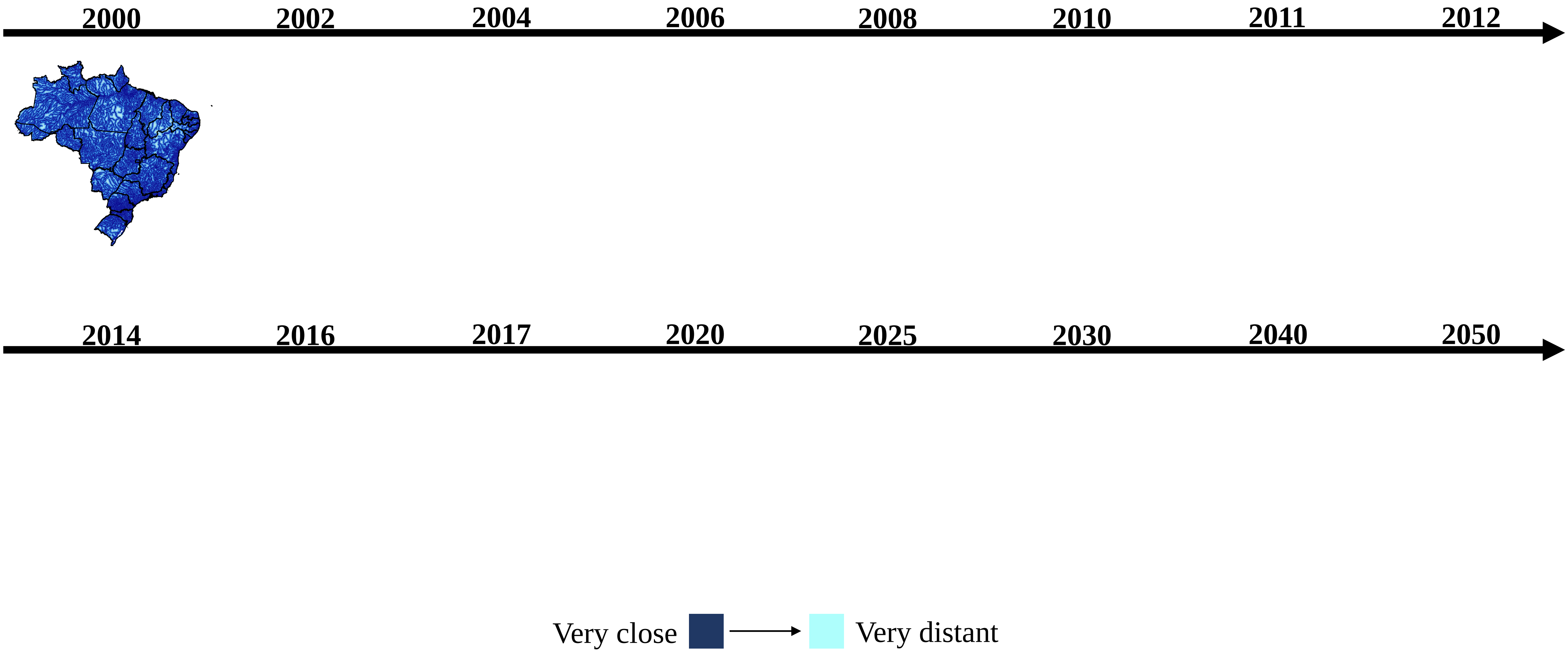

The source of States boundaries is according to IBGE [31]

Scenario independent factors

Railway (Euclidean distance to the railway)

2000 2002 2004 2006 2008 2010 2011 2012

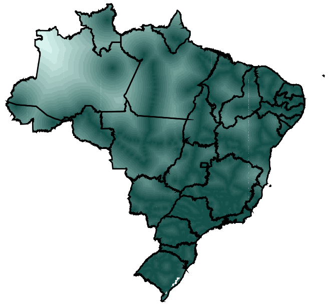

2014 2016 2017 2020 2025 2030 2040 2050

Very close → Very distant

The source of States boundaries is according to IBGE [31]

Scenario independent factors

Urban Centers (Euclidean distance to the urban centers with more than 10 thousand inhabitants)

2000 2002 2004 2006 2008 2010 2011 2012

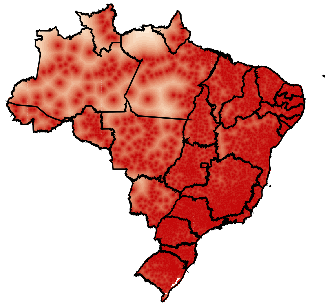

2014 2016 2017 2020 2025 2030 2040 2050

Very close → Very distant

The source of States boundaries is according to IBGE [31]

Scenario independent factors

Urban Centers (Euclidean distance to the urban centers with more than 100 thousand inhabitants)

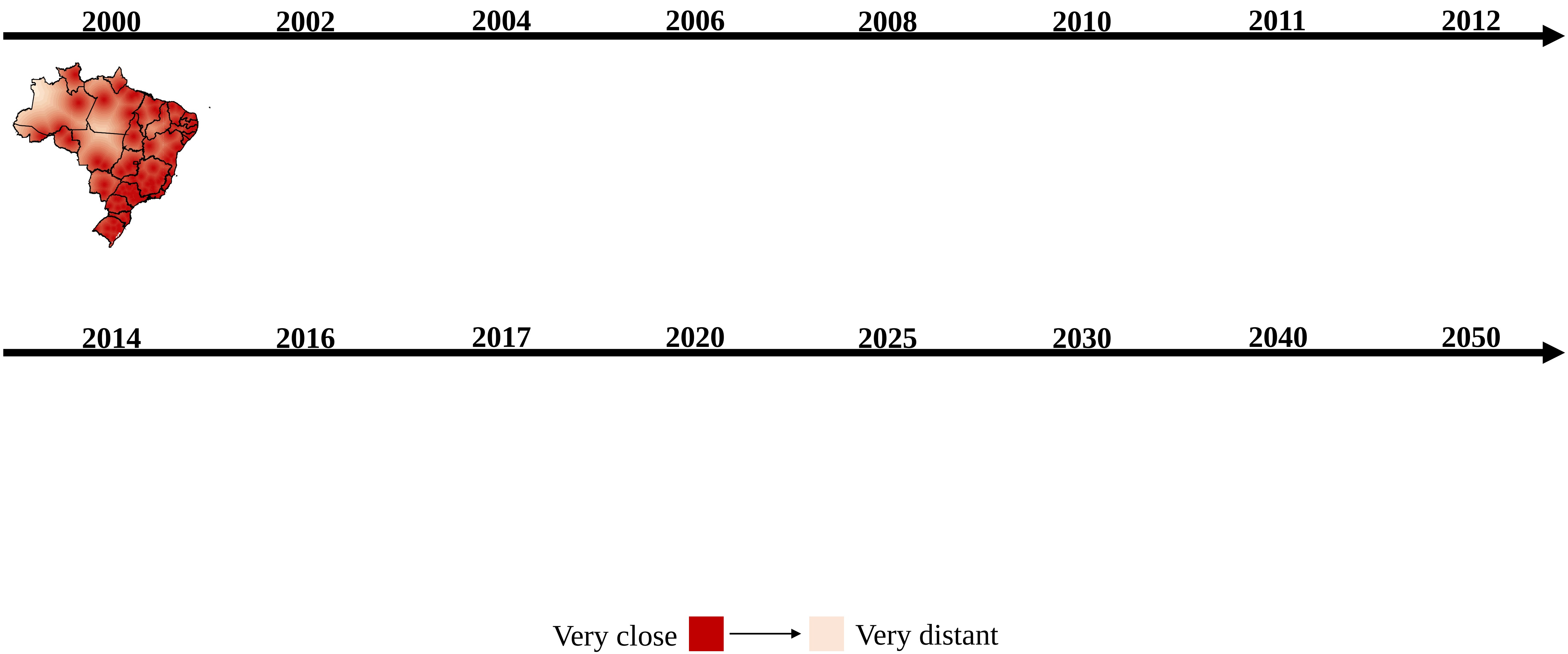

The source of States boundaries is according to IBGE [31]

Scenario independent factors

Agricultural Suitability (Percentage of cell area covered by low agricultural suitability)

2000 2002 2004 2006 2008 2010 2011 2012

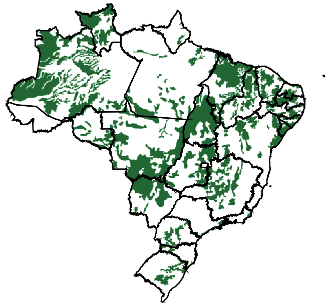

2014 2016 2017 2020 2025 2030 2040 2050

0% → 100%

The source of States boundaries is according to IBGE [31]

Scenario independent factors

Agricultural Suitability (Percentage of cell area covered by medium low agricultural suitability)

2000 2002 2004 2006 2008 2010 2011 2012

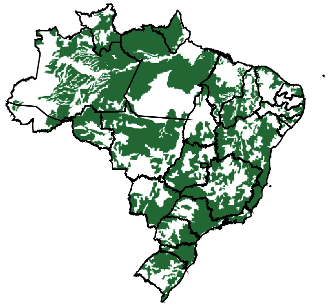

2014 2016 2017 2020 2025 2030 2040 2050

0% → 100%

The source of States boundaries is according to IBGE [31]

Sustainable development scenario (SSP1 RCP 1.9)

MCWDA (Maximum cumulative water deficit)

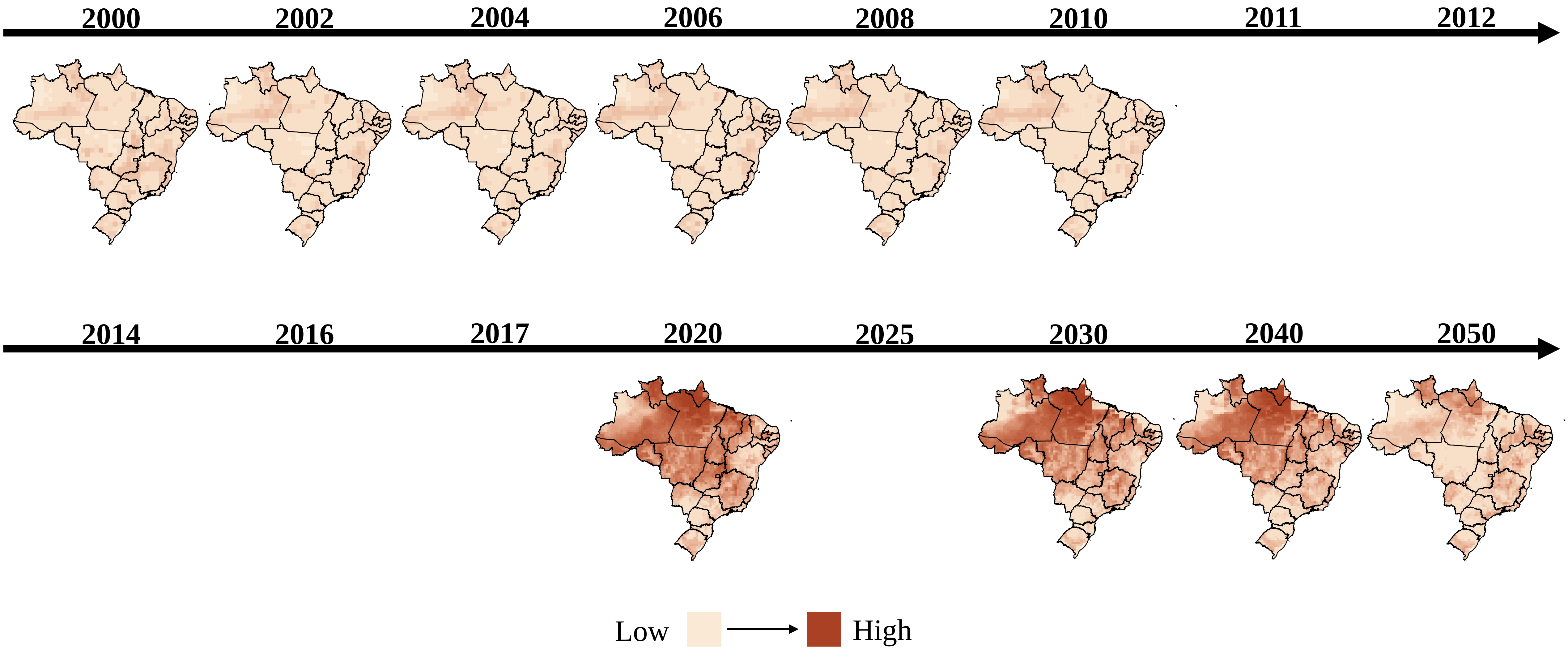

The source of States boundaries is according to IBGE [31]

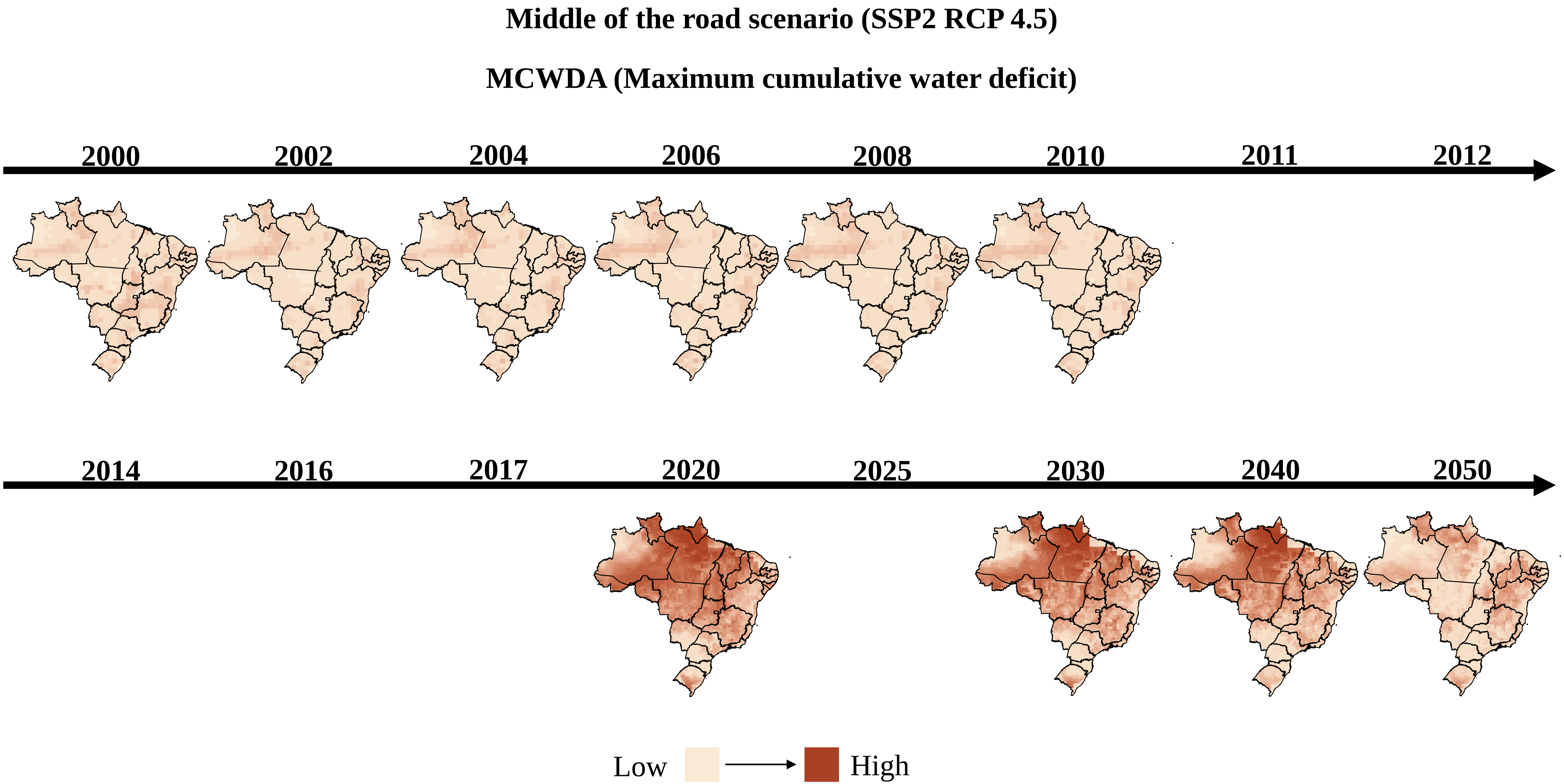

The source of States boundaries is according to IBGE [31]

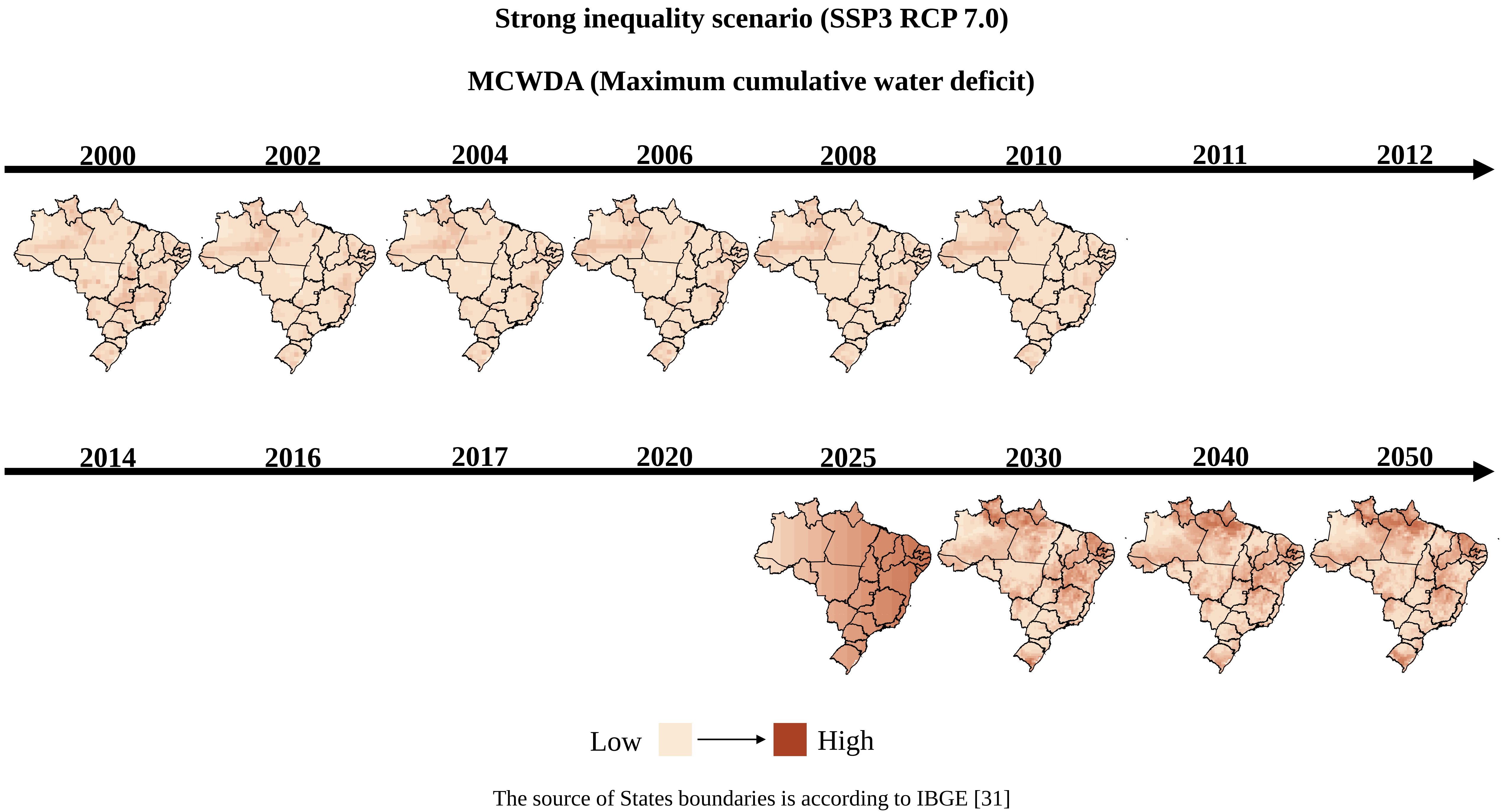
Strong inequality scenario (SSP3 RCP 7.0)
MCWDA (Maximum cumulative water deficit)
2000
2002
2004
2006
2008
2010
2011
2012
2014
2016
2017
2020
2025
2030
2040
2050
Low
High
The source of States boundaries is according to IBGE [31]

Sustainable development scenario (SSP1 RCP 1.9)

Paved Roads (Euclidean distance to the federal and state highways paved)

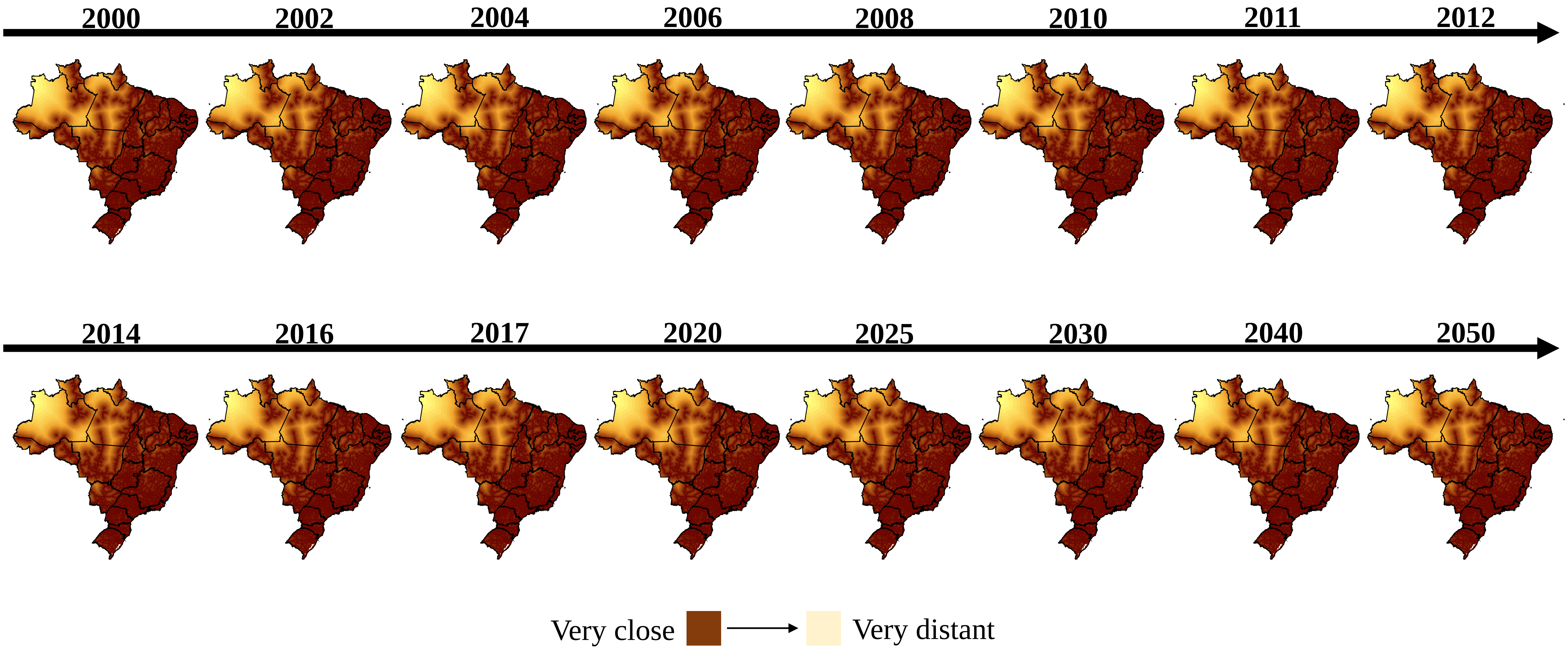

The source of States boundaries is according to IBGE [31]
