# Supplementary material for: New land-use change scenarios for Brazil: Refining global SSPs with a regional spatially-explicit allocation model
Source: PLoS One. 2022 Apr 20;17(4):e0256052. doi: 10.1371/journal.pone.0256052 (PMC9020719; doi:10.1371/journal.pone.0256052)
Supplement: Attachment — Submitted filename: Response_to_Reviewers.pdf [file pone.0256052.s003.pdf]

São José dos Campos, 12 November 2021.

Dear Editor,

We thank the reviewers for their constructive and helpful suggestions. We have made major changes to the manuscript and believe that the manuscript has significantly improved.

Reviewer n. 1

Question: 1 . Please elaborate further the Model performance and the results obtained in Figure 3. As the overall data are projections the validation with existing and simulated data remains a critical parameter that needs to be discussed. If possible please provide more details (per class) and more statistics in this section (probably using a table).

Answer: We appreciate the suggestion. We inserted a table (Table 5) quantifying the performance for each land use and land cover class.

Question: 2. 2.2 Scenario assumptions: from global to regional, and especially the paragraph after line 92. Please provide more details of how you have elaborated the downscaling approach moving from global to regional approach. Why and how did you selected the " SSP1 RCP 1.9, SSP2 RCP 4.5 and SSP3 RCP 7.0—table 3). This is something that needs to be further elaborated and discussed.

Answer: We appreciate the observation and suggestion. We have inserted the storylines for clarity. Line 106-152

Question: 3. Future work and research direction from the authors would be appreciated. What is missing in the current study, are there any steps for the future in terms of improvements of the model.

Answer: Thanks for the suggestion. We have included a paragraph (L318-321) indicating the importance of developing participatory scenarios in the future.

Reviewer n. 2

Question: 1. Please add the definitions of "global", "local", and "balance" here. Take the study area, Brazil, as our example, does the global scale here mean real "global", America, or Latin America? Giving examples of global or local factor can help our readers understand this research problems and focus on the key issues.

Answer: Thanks for the suggestion. We have included a paragraph (L47-57) defining the three scales considered in developing the regional scenarios along with examples of factors/drivers.

Question: 2. The authors can add explanations of their scenarios in the abstract. For example, sustainable development scenario for SPP1 RCP 1.9, middle of the road scenario for SPP2 RCP 4.5, and strong inequality scenario for SPP3 RCP 7.0. Also, what is the number meaning after RCP?

Answer: Thanks for the suggestion. The numbers refer to the forcing for each RCP, the range of radiative forcing values in the year 2100. Radiative forcing is the change in the net, downward

minus upward, radiative flux (expressed in watts per square meter; W m-2) at the tropopause or top of atmosphere due to a change in an external driver of climate change, such as a change in the concentration of carbon dioxide (CO2) or the output of the sun. We entered the radiative forcing values for each RCP. Line 104-108.

Question: 3. This study includes many abbreviations, such as LUCC, LuccMEBR, IBGE, IMAGE, LuccME, INLAND, LR, and PPAS. The authors can provide a list in the appendix.

Answer: Thanks for the suggestion. Done. Line 331

Question:: 4. There is a reference citation error on line 123 page 6.

Answer: Thanks for the observation. Done. Line 173

Question: 5. In table one, the description of some classes are very detailed, but some are more generalized. Is there a specific reason?.

Answer: Thanks for the observation. The descriptions correspond to the classification and original description of the IBGE land use and land cover classes. Furthermore, some of them have a greater diversity of elements in their compositions. We have inserted the related reference to the description. Table 1

Question: 6. For the study method, the authors used a validation matric (equation 5) to test the similarity between the real and simulated maps. Is this the most common method because the reference paper is 1989. Also, what is the range of this NS index and how to explain it?

Answer: Thanks for the observation. Multiscale analysis is crucial for generating land use and land cover change models as well as their validation. Thus, the metrics adopted for validation should establish the level of similarity between simulated and real maps at different resolutions. For this reason, we adopted the multiscale similarity method adapted from Costanza (1989) and Pontius (2002). It allows both location errors in the resolution of the model itself and spatial pattern errors, degrading the resolution of the maps. We have added a reference to Pontius to the manuscript and rewritten the paragraph to clarify the importance of choosing this metric. Line 229-237. As for the range, the NS value can range from 0% (no similarity) to 100% (completely similar).

Question: 7. Please increase resolution of figure 3 and 4.

Answer: About the original Fig. 3, we divided it to allow better visualization. Fig. 3 is now represented in Figs. 4 and 5. As for the original Fig. 4, we grouped it; however, we increased the font size and partially restructured it. We chose these formats to avoid an excessive number of figures.
